# Interaction of Classical Platinum Agents with the Monomeric and Dimeric Atox1 Proteins: A Molecular Dynamics Simulation Study

**DOI:** 10.3390/ijms15010075

**Published:** 2013-12-20

**Authors:** Xiaolei Wang, Chaoqun Li, Yan Wang, Guangju Chen

**Affiliations:** Key Laboratory of Theoretical and Computational Photochemistry, Ministry of Education, College of Chemistry, Beijing Normal University, Beijing 100875, China; E-Mails: wxl20112115@sina.com (X.W.); lichaoqun_210@163.com (C.L.)

**Keywords:** molecular dynamics simulations, molecular mechanics Poisson-Boltzmann surface area (MM-PBSA), Atox1 protein, cisplatin, transplaitn, oxaliplatin, interaction mechanism

## Abstract

We carried out molecular dynamics simulations and free energy calculations for a series of binary and ternary models of the cisplatin, transplatin and oxaliplatin agents binding to a monomeric Atox1 protein and a dimeric Atox1 protein to investigate their interaction mechanisms. All three platinum agents could respectively combine with the monomeric Atox1 protein and the dimeric Atox1 protein to form a stable binary and ternary complex due to the covalent interaction of the platinum center with the Atox1 protein. The results suggested that the extra interaction from the oxaliplatin ligand–Atox1 protein interface increases its affinity only for the OxaliPt + Atox1 model. The binding of the oxaliplatin agent to the Atox1 protein might cause larger deformation of the protein than those of the cisplatin and transplatin agents due to the larger size of the oxaliplatin ligand. However, the extra interactions to facilitate the stabilities of the ternary CisPt + 2Atox1 and OxaliPt + 2Atox1 models come from the α1 helices and α2-β4 loops of the Atox1 protein–Atox1 protein interface due to the cis conformation of the platinum agents. The combinations of two Atox1 proteins in an asymmetric way in the three ternary models were analyzed. These investigations might provide detailed information for understanding the interaction mechanism of the platinum agents binding to the Atox1 protein in the cytoplasm.

## 1. Introduction

The classical anticancer platinum agents, such as cisplatin [*cis-*diamminedichloroplatinum (II)], transplatin [*trans*-diamminedichloroplatinum (II)] and oxaliplatin [1,2-diaminocyclohexane- oxalateplatinum (II)], were used widely in clinical therapy effectively against several common types of tumors [1–6], such as ovarian, testicular and lung cancer tumors. The mechanism of the platinum agents against tumor is thought to be that the Pt (II) center attacks DNA through the contacts of covalent bonds with the N7 atoms of guanines of the DNA molecule in order to prevent the replication and transcription of DNA, consequently to lead to the cell apoptosis [7–10]. In cancer treatment, the platinum agents transiting the nuclear membranes and reacting with DNA play a key role in improving the therapeutic efficacy. However, the efficacy of these platinum agents is restricted by the reduced uptake to the cytoplasm and increased efflux, consequently decreasing the possibility of the DNA target. In fact, the platinum agents can react rapidly with some macromolecules in the cytoplasm, especially thiol compounds, and become invalid before entering cell nucleus [11,12]. Therefore, the studies on the resistance mechanism of the platinum agents and the interactions between the platinum agents and the biomacromolecules have become a hot topic in recent years.

To date, many studies have proposed that copper (Cu) transporting proteins are related with both uptake and efflux of platinum agents in the cytoplasm [13–16]. For example, it has been reported that the copper transporter 1 (Ctrl) protein, one of the copper influx transporting proteins, is responsible for platinum agent uptake [17,18], whereas the copper transporting ATPases ATP7A and ATP7B proteins (the Menkes and Wilson disease proteins, respectively) are involved in the platinum agent efflux from cell [19,20]. Each of the ATP7A and ATP7B proteins has six *N*-terminal metal-binding domains (MBDs) with similar structures. Each metal-binding domain has a ferredoxin like fold and a conserved metal-binding site, MetX_1_CysX_2_X_3_Cys (*X_i_* = any residue), in a solvent-exposed loop, which contributes to the metal transporting. Interestingly, the NMR solution structure of the chaperone Atox1 (antioxidant-1) protein reported by Bertini and co-workers in 2004 presented that this protein also has a ferredoxin like fold and a conserved metal-binding site, the structures of which are similar to those in the ATP7A and ATP7B proteins [21]. Therefore, the Atox1 protein is thought to be a metal transporting protein, and be able to interact with the platinum agent to influence its accumulation in the cytoplasm. The studies of the Atox1 mutations in mice reported by Safaei and co-workers suggested that the Atox1 protein can bind to the cisplatin agent [22]. Subsequently, the X-ray crystal structures of the cisplatin agent + Atox1 protein complexes were reported by Rosenzweig and co-workers in 2009 [23], which present the combination of a platinum agent with an Atox1 protein or an Atox1 dimer. The Atox1 protein with 68 residues consists of a compact β1α1β2β3α2β4 structure with the conserved metal-binding sites, Met10Thr11Cys12Gly13Gly14Cys15, located at the solvent-exposed β1-α1 loop that is a linker of the β1 strand and the α1 helix. The binary complex is composed by a single Atox1 protein and a platinum ion that is coordinated by two S atoms of Cys12 and Cys15 at the metal-binding sites of the Atox1 protein [23]. In the ternary structure, the two Atox1 proteins are linked by the platinum center of the cisplatin agent at the same binding sites of residues Cys15 [23]. In addition, the NMR experimental results reported by Natile and co-workers presented that the cisplatin + Atox1 derivative could be characterized by having a slightly strained chelate ring, affording a free position for sequential binding of an Atox1 protein [24]. Recently, Schaffner, Wittung-Stafshede and co-workers have reported that the Atox1 levels in cells have an evident influence on the cisplatin agent sensitivity [25,26]. Interestingly, it has been found that some cancer cell lines with the higher levels of the Atox1 protein have correspondingly higher resistance to the cisplatin agent than the cell lines with lower levels of the Atox1 protein [27,28], which means that the binding of cispaltin agent to the Atox1 protein can resist the activity of the cisplatin agent in the cancer treatment. Moreover, it has been reported through experimental studies on the platinum anticancer agents, such as cisplatin, transplatin, carboplatin, oxaliplatin, pyriplatin agents, *etc.*, that these platinum agents binding to the Atox1 protein can promote the Atox1 protein unfolding followed by the aggregation of the proteins with the increase of the incubation time [29]. This result indicated that the Atox1 protein may act as a hijacker of the platinum agents to prevent the platinum agents crossing the nuclear membrane and interacting with a consensus sequence of DNA. However, the details of the mechanism of the interaction between the platinum agents and the Atox1 protein have not been fully understood. Theoretical investigations devoted to this field are also lack at the atomic level so far.

To elucidate the interaction mechanism of the platinum agents and the Atox1 protein, we select the three classical platinum agents, cisplatin, transplatin and oxaliplatin, as the agent examples, respectively binding to a monomeric Atox1 protein or a dimeric Atox1 protein to build six binary and ternary models. The molecular dynamics (MD) simulations and free energy calculations for these models were carried out to investigate the interaction mechanism of the platinum agents and the Atox1 proteins, the effect of ligand sizes and cis/trans conformations of platinum agents on the interaction properties and the protein deformation. This study may provide valuable insights into detailed mechanisms of uptake and efflux of platinum agents in the cytoplasm at the atomic level.

## 2. Results

**Figure 1.**
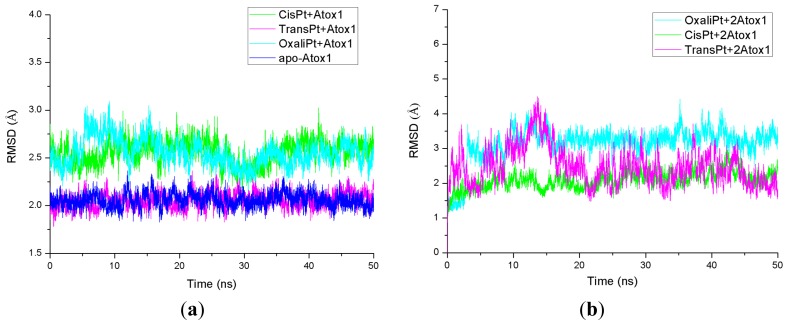
Root-mean-square deviation (RMSD) values of all backbone atoms with respect to the corresponding starting structures for the simulations of (**a**) the apo-Atox1 (**light blue**), CisPt + Atox1 (**green**), TransPt + Atox1 (**magenta**) and OxaliPt + Atox1 (**cyan**) models; and (**b**) the CisPt + 2Atox1 (**green**), TransPt + 2Atox1 (**magenta**) and OxaliPt + 2Atox1 (**cyan**) models.

The root-mean-square deviation (RMSD) values of all backbone atoms referenced to the corresponding starting structures over all trajectories for the apo-Atox1, CisPt + Atox1, TransPt + Atox1, OxaliPt + Atox1, CisPt + 2Atox1, TransPt + 2Atox1 and OxaliPt + 2Atox1 models were examined to determine if each system had attained equilibrium and are shown in Figure 1. It is often considered that small RMSD values of a simulation indicate a stable state of the system. For example, the average RMSD value of the backbone atoms is 2.1 Å for the CisPt + 2Atox1 model, which suggests that no significant structural drift from the corresponding crystal structure occurred in this model. It can be further seen from Figure 1 that each model has reached equilibrium after 30 ns, and the corresponding energy was found to be stable during the last 20 ns simulation. Moreover, the calculated conformational stability free energies of the binary models as a function of time, during the last 20 ns simulation, were also analyzed and are shown in Figure S1 for the CisPt + Atox1, TransPt + Atox1 and OxaliPt + Atox1 models. The computed trends showed a favorable stability with a small fluctuant during each simulation. Therefore, the trajectory analysis for these systems has extracted the equilibrated conformations between 30 and 50 ns of simulation time, recording 10,000 snapshots at every 2 ps time-interval of each trajectory.

### 2.1. The Characteristics of Platinum Agents Combining with a Monomeric Atox1 Protein

#### 2.1.1. The Structure Characteristics of Platinum + Atox1 Complexes

**Figure 2.**
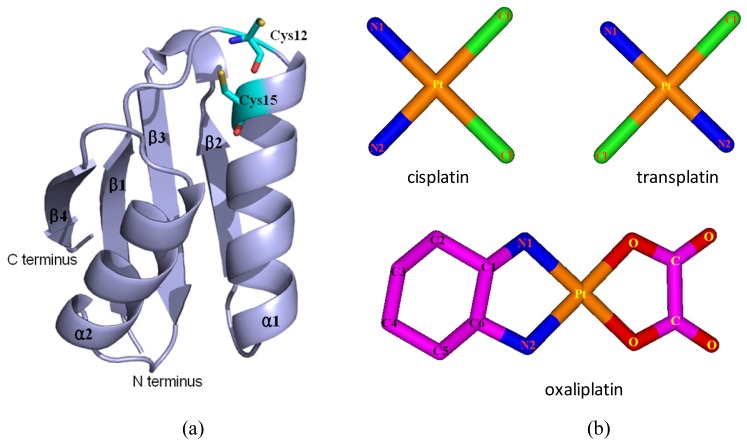
The structures of (**a**) the apo Atox1 protein; and (**b**) the cisplatin(II), transplatin(II) and oxaliplatin(II) agents. (Pt: orange; N: deep blue; C: cyan/magenta; S: yellow; O: red; Cl: green).

**Figure 3.**
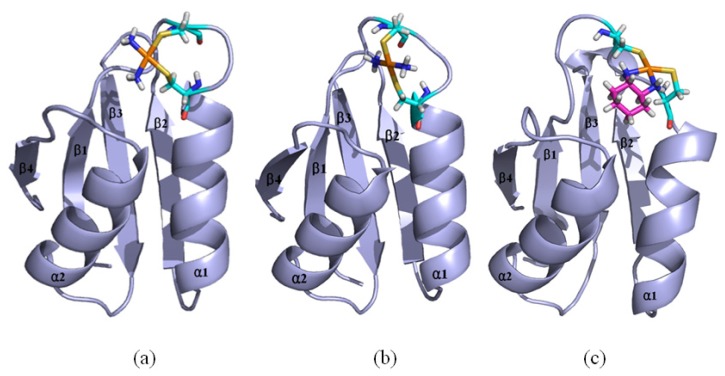
The average structures of (**a**) the CisPt + Atox1 model; (**b**) the TransPt + Atox1 model; and (**c**) the OxaliPt + Atox1 model (Pt: orange; N: deep blue; C: cyan/magenta; S: yellow; O: red; H: white).

**Figure 4.**
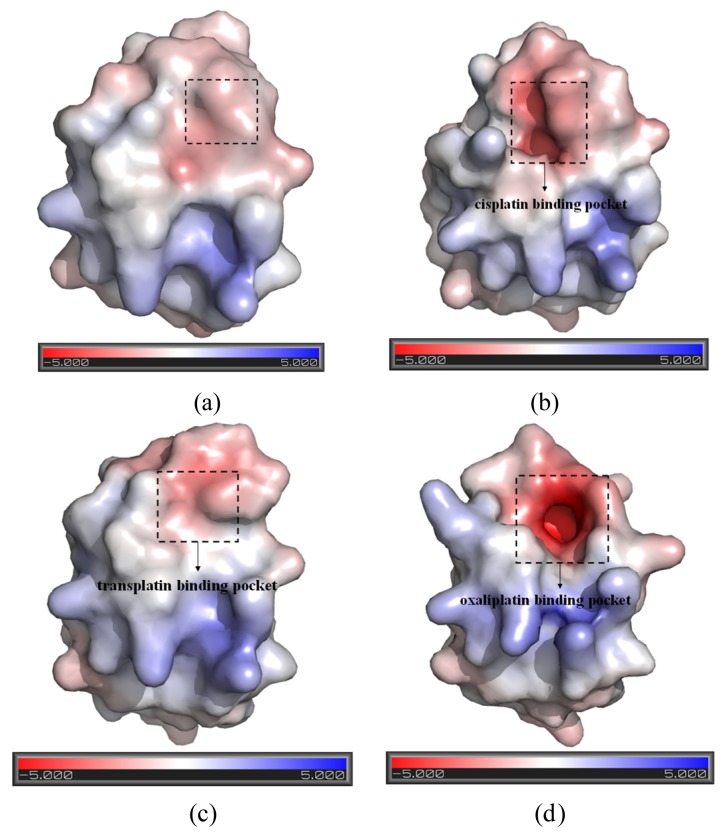
The representation of the pockets and the electrostatic surface potential of (**a**) the apo-Atox1 model; (**b**) the CisPt + Atox1 model; (**c**) the TransPt + Atox1 model; and (**d**) the OxaliPt + Atox1 model (**red**: negative charge; **blue**: positive charge).

**Figure 5.**
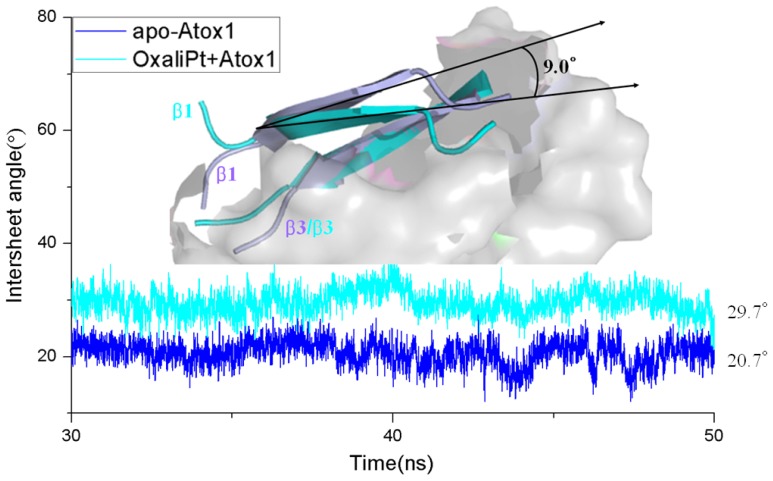
The time-dependence of the intersheet angles between the β1 and β3 strands of the Atox1 protein for the apo-Atox1 (**light blue**) and OxliPt + Atox1 (**cyan**) models. The inset is the visual superposition for the average structures of the β1 and β3 strands for the two models.

Based on the previous studies of platinum agents, the platinum center in the cisplatin or transplatin agent connects two ammonias and two chlorides with the cis or trans conformation [30,31]. However, the platinum center in the oxaliplatin agent is coordinated by two nitrogens from 1,2-diaminecyclohexane and two oxygens from the oxalate. It is believed that the oxaliplatin agent loses the oxalate group, and retains its 1,2-diaminocyclohexane moiety in cell environments [32]. Therefore, each platinum center in all the three platinum agents has two active binding sites for combining with the Atox1 protein. The apo Atox1 protein molecule based on the NMR solution structure [21] consists of one α-helical layer of α2, α1 with the angle of 46.6° between the two α helices, and one β-sheet layer of β4, β1, β3, β2 arranging orderly from left to right, and is shown in Figure 2 with the total connection order of β1-α1-β2-β3-α2-β4. The two layers in the protein are almost parallel to each other. The metal-binding sites in this protein are the residue Cys12 located at the solvent-exposed β1-α1 loop, and the residue Cys15 located at the α1 *N*-terminal that connects the β1-α1 loop. To investigate the structural characteristics of the platinum agents binding to the Atox1 protein, the CisPt + Atox1, TransPt + Atox1 and OxaliPt +Atox1 models were simulated, and the average structures extracted from the trajectories are shown in Figure 3. It is demonstrated that each platinum agent with the platinum center coordinating to the two residues Cys12/Cys15 is located at the middle of the β1-α1 loop and the α2-β4 loop that is a linker of the α2 helix and the β4 strand in the Atox1 protein. Due to the differences of ligands in the three platinum agents, the ligand plane parallels to the β-sheet in the CisPt + Atox1 model, while is almost perpendicular to that in the TransPt + Atox1 model and forms a certain angle with the β-sheet in the OxaliPt + Atox1 model. As expected, the oxaliplatin agent with a large ligand binding to the Atox1 protein leads to the formation of a large binging pocket with the size of 82.6 Å^2^/61.5 Å^3^ and the increase of the intersheet angle of β1 and β3 strands by ~9° in the OxaliPt + Atox1 model over the cisplatin and transplatin agents (see Figures 4 and 5 and Discussion Section). Interestingly, the binding of the platinum agent to the Atox1 protein causes the unfolding of some residues at some α helices and β strands, which predicts the deformation of the Atox1 protein (see Discussion Section). In particular, the oxaliplatin agent triggers the extensive deformation of the Atox1 protein due to its large ligand size.

#### 2.1.2. Free Energy and Interaction Analysis of Platinum + Atox1 Complexes

To address the affinity of three platinum agents to the Atox1 protein, the binding free energy calculations for the CisPt + Atox1, TransPt + Atox1 and OxaliPt + Atox1 models have been carried out by using MM-PBSA methodology based on the standard molecular dynamics simulations. Because each platinum center in the three agents forms two equivalent covalent bonds with two same residues Cys12 and Cys15, such covalent interactions were ignored in the binding free energy calculations. All energy terms and the total binding free energies for the non-covalent interactions in these models have been given in Table 1. It can be seen that the ligand of the oxaliplatin agent binding to the Atox1 protein with the binding free energy of −4.13 kcal·mol^−1^ in the OxaliPt + Atox1 model is energetically favorable, which proposes that this extra interaction coming from the oxaliplatin ligand with the Atox1 protein will be helpful to efficiently weaken the DNA-targeted activity of the platinum agent in the nucleus. However, the calculated binding free energies of 5.28 and 12.41 kcal·mol^−1^ in the CisPt + Atox1 and TransPt + Atox1 models predict the insignificant interaction contribution by the cisplatin/transplatin ligand–Atox1 protein interface.

**Figure 6.**
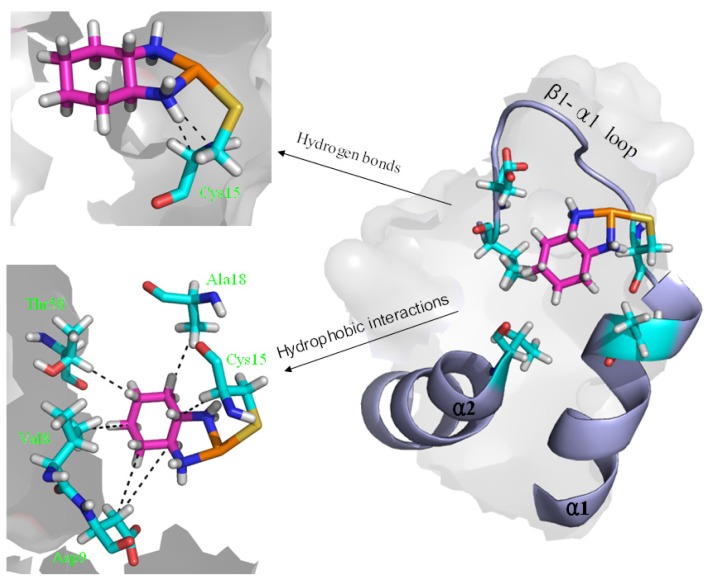
The scheme of the extra hydrogen bond and hydrophobic interactions for the OxaliPt + Atox1 model. (Pt: orange; N: deep blue; C: cyan/magenta; S: yellow; O: red; H: white).

To explore the affinity property between the oxaliplatin ligand and the protein, the occupancies of occurrences of dominant hydrogen bonds at the oxaliplatin ligand–Atox1 protein interface were analyzed by calculating the percentages of simulation times, and are shown in Table 2 and Figure 6. The criteria for an intermolecular/intramolecular hydrogen bond was a donor-acceptor distance of <3.5 Å and a donor-proton-acceptor angle of >120° [33,34]. The formations of extra hydrogen bonds between the N–H groups/N atom of the oxaliplatin ligand and the C atoms/C–H group of the residue Cys15 of the β1-α1 loop in the Atox1 protein respectively with the occupancies of the 176.6% and 30.6% have been found, which supports the calculated affinity for the oxaliplatin ligand–Atox1 protein interface in the OxaliPt + Atox1 model. To further examine the differences of interactions between the platinum ligand and the protein, hydrophobic interactions, which are defined as a distance shorter than 4.5 Å between carbon atoms with sp^3^ hybridization, were also analyzed by calculating the percentages of simulation times for the OxaliPt + Atox1 model and are shown in Table 2 and Figure 6 if they occurred in more than 50% of the snapshots, making percentage numbers > 100 possible [35]. It can be seen that the OxaliPt + Atox1 model spends the total occupancies of 742.5% in the formations of hydrophobic interactions between the oxaliplatin ligand and the Atox1 protein. For example, the formations of hydrophobic interactions between the diaminocyclohexane of the oxaliplatin agent and the residues Cys15 of the β1-α1 loop, Ala18 of the α1 helix, Thr58 of the α2-β4 loop in the Atox1 protein are maintained respectively with the occupancies of 197.2%, 76.3% and 50.9% for the OxaliPt + Atox1 model. As expected, the existence of such extra hydrogen bonds and hydrophobic interactions leads to the ligand plane of the oxaliplatin agent inlaying into the binding pocket, consequently causes the considerable deformation of the Atox1 protein. In addition, the electrostatic surface analysis was carried out and is shown in Figure 4. The analysis results reveal that the oxaliplatin binding pocket (including the residues Val8, Asp9, Cys12, Gly13, Gly14 of the β1-α1 loop, Ile33 of the β2 strand and Lys38 of the β3 strand) in the OxaliPt + Atox1 model has a stronger negative surface charge than cisplatin/transplatin binding pocket in the CisPt + Atox1/TransPt + Atox1 model, which may favor the binding of positive-charged oxaliplatin agent to the negative-charged pocket residues in the Atox1 protein. Summarily, the simulation analysis indicates that all the three platinum agents can respectively combine with a monomeric Atox1 protein to form a stable binary complex due to the covalent interaction of the platinum center with the binding sites in the Atox1 protein. The interaction analysis supports the calculated affinities of platinum agents to the Atox1 protein, and further indicates that the oxaliplatin agent can strongly bind to the Atox1 protein in the cytoplasm to block its transport to DNA.

#### 2.1.3. Dynamical Fluctuation Analysis for Platinum + Atox1 Complexes

**Figure 7.**
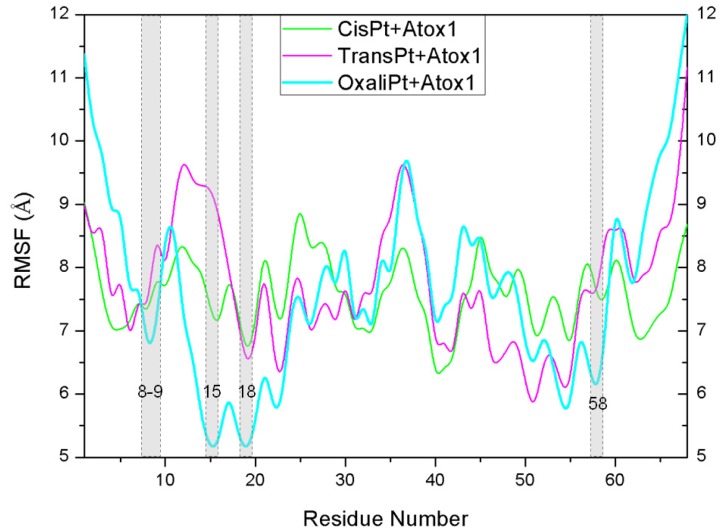
The fluctuations of residues for the CisPt + Atox1 (**green**), TransPt + Atox1 (**magenta**) and OxaliPt + Atox1 (**cyan**) models.

To address the interactions of the platinum agents with the Atox1 protein and its conformation changes induced by the platinum agents binding via the residue position changes, the dynamics of every residue were determined and interpreted by residue fluctuations. The RMSF values were analyzed and are shown in Figure 7 for the CisPt + Atox1, TransPt + Atox1 and OxaliPt + Atox1 models. It can be observed that the hydrogen bonds and hydrophobic interactions between the platinum ligand and the Atox1 protein can be directly linked to the changes of RMSF values in the fluctuation pattern. Most interesting of all, the extra hydrogen bonds between the oxaliplatin ligand and the residue Cys15 of the β1-α1 loop of the Atox1 protein, and the hydrophobic interactions between the oxaliplatin ligand and the residues Cys15 of the β1-α1 loop, Ala18 of the α1 helix, Thr58 of the α2-β4 loop cause the decrease of the corresponding fluctuations, which represents the stability of corresponding contact sites and the large affinity of the oxaplatina ligand to the Atox1 protein. These results are consistent with the hydrogen bonds and hydrophobic interactions analyses discussed above.

### 2.2. The Characteristics of Platinum Agents Combining with a Dimeric Atox1 Protein

**Figure 8.**
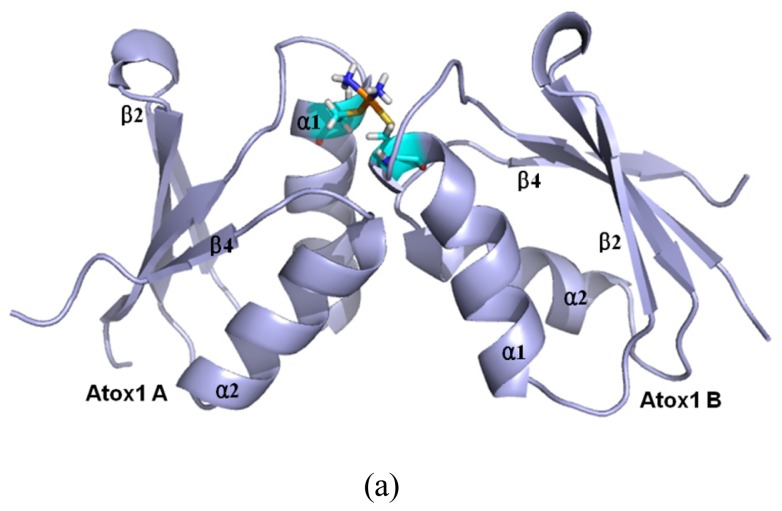

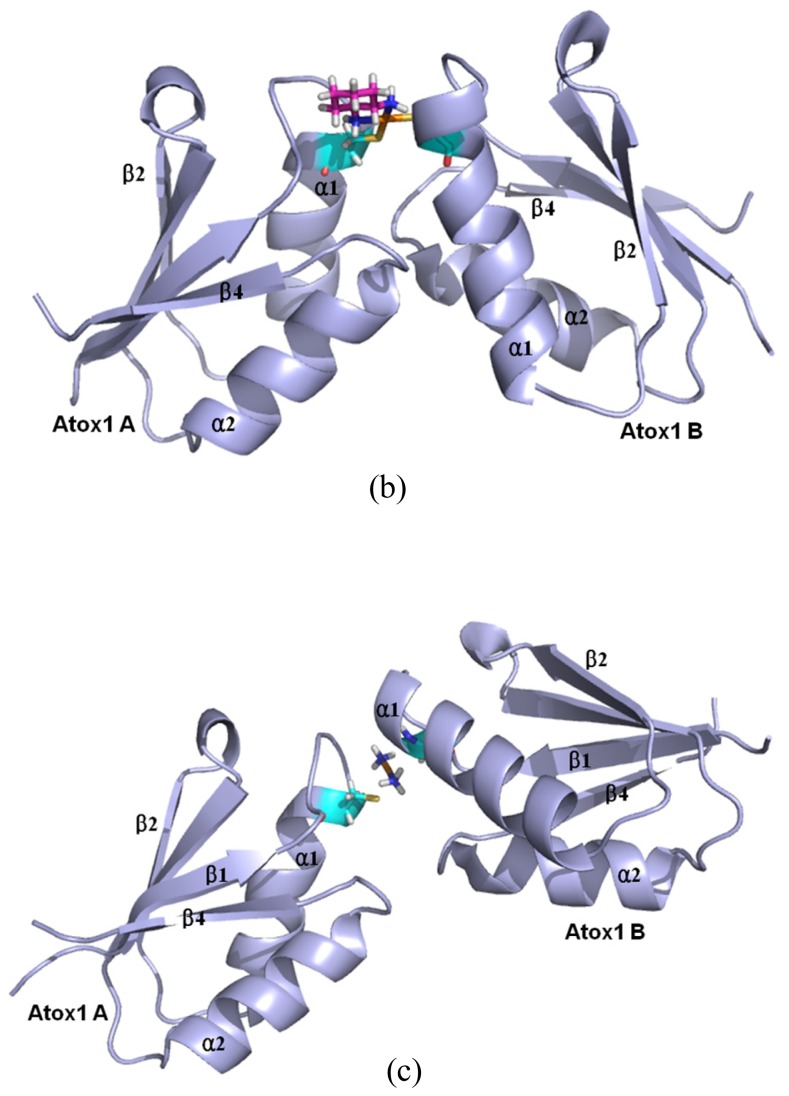
The average structures of (**a**) the CisPt + 2Atox1 model; (**b**) the OxaliPt + 2Atox1 model; and (**c**) the TransPt + 2Atox1 model (Pt: orange; N: deep blue; C: cyan/magenta; S: yellow; O: red; H: white).

Based on the previous experimental study, a platinum agent can combine with not only a monomeric Atox1 protein but also a dimeric Atox1 protein [23]. In particular, it has been observed from the previous experiments that the metal (Cu, Cd)-bridged Atox1 dimer plays a key role in metal transfer between the Atox1 protein and its target MBDs of ATP7B [36,37]. To address the characteristics of an Atox1 dimer bound by the platinum agents, we performed the MD simulations for the three cisplatin, transplatin and oxaliplatin agents binding to the Atox1 dimer, as three models CisPt + 2Atox1, TransPt + 2Atox1 and OxaliPt + 2Atox1. The root-mean-square deviation (RMSD) values of all backbone atoms for the three models were examined and are shown in Figure 1b. The average structures extracted from the trajectories for the three models are shown in Figure 8. It can be seen that the platinum center in the platinum agents binds to the residue Cys15 at each α1 helix of the Atox1 dimer with the ligand plane of each platinum agent located at the middle of the Atox1 dimer. The ligand plane of the cisplatin or oxaliplatin agent in the CisPt + 2Atox1 or the OxaliPt + 2Atox1 model is towards the outside of the Atox1 dimer due to its cis conformation. The two α-helical layers of the Atox1 dimer with the two outer β-sheet layers face each other. The two Atox1 proteins with the angle of ~36° between two α1 helices in the CisPt + 2Atox1 and OxaliPt + 2Atox1 models are close to each other over those with the angle of ~85° in the TransPt + 2Atox1 model being away from each other due to the trans conformation of platinum agent. In particular, such trans conformation in the transplatin agent causes the larger relative rotation angle of two proteins by about 70° over the cis conformation for the other two platinum agents (see Figure S2 and Discussion Section). Moreover, the two Atox1 proteins in each ternary model present an asymmetric dimeric structure, which reproduces the experimental suggestion [23]. To understand the non-covalent interactions between the platinum ligand and the Atox1 dimer and between the two Atox1 proteins, the binding free energy calculations and the interaction analysis for the CisPt +2Atox1, TransPt + 2Atox1 and OxaliPt + 2Atox1 models were evaluated and are shown in Tables S1, S2 and Figure S3. It can be seen that the binding free energies between the platinum ligands and the Atox1 dimers, except for the covalent interaction between the platinum center and the binding sites in the Atox1 dimer, for the CisPt + 2Atox1, TransPt + 2Atox1 and OxaliPt + 2Atox1 models are positive values, which predicts no extra interaction between the platinum ligand and the Atox1 dimer. However, the binding free energies between the Atox1 A protein and Atox1 B protein in the CisPt +2Atox1 and OxaliPt + 2Atox1 models are −3.57 and −5.06 kcal·mol^−1^, respectively, which presents that such interaction can stabilize the ternary cisplatin + 2Atox1 and oxaliplatin + 2Atox1 complexes. As expected, the formations of hydrogen bonds between the α1 helix, the α2-β4 loop of the Atox1 A protein and those of the Atox1 B protein in the CisPt + 2Atox1 and OxaliPt + 2Atox1 models respectively with the occupancies of 89.2% and 147.9% are found (see Table S2 and Figure S3). The hydrophobic interaction analysis predicts that the CisPt + 2Atox1 and OxaliPt + 2Atox1 models respectively spend the total occupancies of 703.9% and 767.2% in the formations of hydrophobic contacts between the two Atox1 proteins (see Table S2 and Figure S3). For example, the formations of hydrophobic interactions between the residue Gly59 of the α2-β4 loop in the Atox1 A protein and the residue Ala18 of α1 helix in the Atox1 B protein for the CisPt + 2Atox1 and OxaliPt + 2Atox1 models are maintained with the occupancies of 96.9% and 166.2% respectively. However, such interactions are insignificant for the TransPt + 2Atox1 model due to the calculated positive binding free energy for the Atox1 protein–Atox1 protein interface. Summarily, each of three platinum agents can combine with a dimeric Atox1 to form a stable ternary complex due to the covalent interaction of the platinum center–Atox1 protein interface. In particular, the cis conformation in the platinum agent favors the interaction of the protein–protein interface in the platinum + 2Atox1 complex.

## 3. Discussion

### 3.1. The Structure Analysis of Platinum Agents Combining with the Monomeric and Dimeric Atox1 Protein

**Figure 9.**
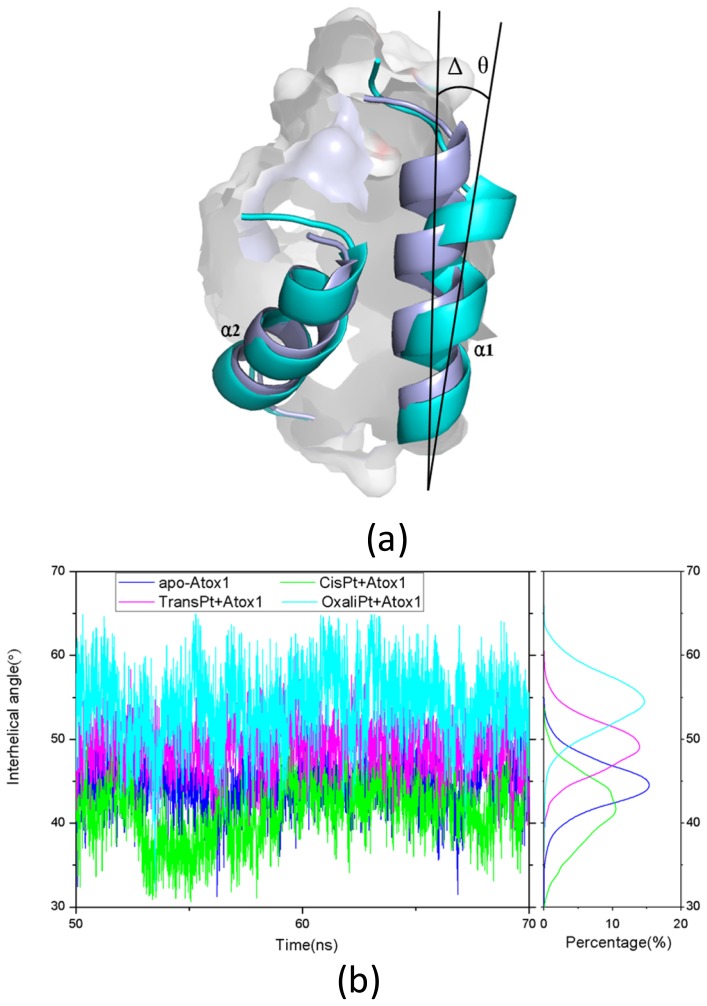
(**a**) The change of the interhelical angle (Δθ) between the α1 and α2 helices for the apo-Atox1 model (**light blue**) and the binary models (**cyan**); and (**b**) The time-dependence of the interhelical angles along with the respective integrated distributions for the apo-Atox1 (**light blue**), CisPt + Atox1 (**green**), TransPt + Atox1 (**magenta**) and OxaliPt + Atox1 (**cyan**) models.

Base on the average structure and the trajectory analysis, the distinct differences of structures in the CisPt + Atox1, TransPt + Atox1, and OxaliPt + Atox1 models have been found. Due to the protein binding sites located at the α1 *N*-terminal and the β1-α1 loop for the three models, the centroid distances between the β1 strand and α1 helix have been analyzed to address the structural differences. It can be seen that such distance change from the average value of 13.5 Å in the apo-Atox1 model to 14.0 Å only in the OxaliPt + Atox1 model. However, such change is insignificant for the CisPt + Atox1 and TransPt + Atox1 models. In particular, in the OxaliPt + Atox1 model, the diaminocyclohexane plane of the oxaliplatin agent forms a certain angle with the β-sheet, and tilts up. The ligand of oxaliplatin agent is located at the α1 *N*-terminal and the α2 *C*-terminal, which causes the movement of α1 helix close to the β-sheet and away from the α2 helix, itself bend of α1 helix, and the increase of intersheet angle of β1 and β3 strands by ~9° (see Figure 5) compared with that in the apo-Atox1 model. While the ligand plane is parallel to the β-sheet in the cisplatin agent of the CisPt + Atox1 model, and is perpendicular to that in the transplatin agent of the TransPt + Atox1 model. However, the binding of the cisplatin and the transplatin agents to the Atox1 protein in the CisPt + Atox1 and TransPt + Atox1 models does not cause any change of the intersheet angle between the β1 and β3 strands over that in the apo-Atox1 model due to the small sizes of the ligands in the two agents. To investigate the structural characteristics of the Atox1 protein, the interhelical angles of the α1 and α2 helices in the apo-Atox1, CisPt + Atox1, TransPt + Atox1 and OxaliPt + Atox1 models have been quantitatively examined over the simulation times by using the program INTERHLX and are shown in Figure 9. The average interhelical angle values are 46.6°, 41.6°, 48.5° and 54.1° for the apo-Atox1, CisPt + Atox1, TransPt + Atox1 and OxaliPt + Atox1 models, respectively, which presents the oxaliplatin agent causes larger extension of the α1 and α2 helices with the largest interhelical angle value. Interestingly, the large arc formed by the loop region (consisting of the residues Asp9-Gly14) linking α1 and β1 in the CisPt +Atox1 model causes the decrease of the interhelical angle of α1 and α2 compared with that in the apo-Atox1 model. Oppositely, the small arc formed by the same loop region in the TransPt + Atox1 and OxaliPt + Atox1 models causes its increase. It’s evident that the residues Gly13/Gly14 of α1 helix, Val62 of β4 strand for the CisPt + Atox1 model, the residues Gly13/Gly14 for the TransPt + Atox1 model, and the residues Gly13-Cys15/Val62 for the OxaliPt + Atox1 model are rearranged from the α helix or β strand structure to the loop conformations due to the platinum agents binding to the residues Cys12 and Cys15. Moreover, the destruction of the hydrogen bonds between the residues Gly13 and Cys15 of the α1 helix in the Atox1 protein from total occupancies of simulation times of 35.3% in the apo-Atox1 model to the occupancies of 11.2% and 0% in the TransPt + Atox1 model and CisPt + Atox1/OxaliPt + Atox1 models, respectively, also supports the deformation of the Gly13-Cys15 from the α helix to the loop conformations. These results presented that the oxaliplatin agent binding causes an extensive unfolding of the Atox1 protein due to more unfolding residues in the binary complex. To further investigate the protein unfolding induced by the platinum agents binding, the CASTp program was employed to calculate the size and shape of platinum agents binding site pockets. Such analysis was able to provide a comprehensive and detailed quantitative characterization of protein surface pockets and protein deformation [38–40]. It can be seen from Figure 4 that the binding site pocket sizes are 0 Å^2^/0 Å^3^, 60.3 Å^2^/45.5 Å^3^, 44.9 Å^2^/32.2 Å^3^ and 82.6 Å^2^/61.5 Å^3^ for the apo-Atox1, CisPt + Atox1, TransPt + Atox1 and OxaliPt + Atox1 models, respectively, which presented that the platinum agents binding to the Atox1 protein cause the formation of the platinum agents binding pocket around the residues Val8, Asp9, Cys12, Gly13, Gly14 and Cys15 *etc.* in the β1-α1 loop of the protein, consequently the deformation of the original Atox1 structure. Specially, the large ligand size of oxaliplatin agent plays a key role in the formation of a largest binding pocket in the Atox1 protein. Summarily, based on the analysis for the unfolding residues and the pocket sizes around the binding sites of Atox1, the order of the extent of the Atox1 unfolding is the oxaliplatin, cisplatin and transplatin agents, which is consistent with the previous experimental result reported by Wittung-Stafshede and co-workers [29]. In particular, the oxaliplatin agent binding to the Atox1 protein is able to cause the larger deformation of the protein with the formation of large binding pockets and the more extensions of the interhelical angle of α1 and α2 helices, and the increase of the centroid distances of the β1 strand and α1 helix, compared with other two cisplatin and transplatin agents. Furthermore, the large deformation of the Atox1 protein induced by the oxaliplatin agent binding favors the affinity of the platinum agent to the protein. Moreover, due to Atox1 functionally acting as a Cu transport protein, it is possible that Cu and Pt bind to two Cys residues in the Atox1 protein simultaneously, which was showed *in vitro* as reported previously [25]. It is suggested that a Cu ion and a platinum agent simultaneously binding to two Cys residues may cause the more looseness of the binding loop region of Atox1 due to the appearance of an additional Cu ion in the Atox1 protein, resulting in the large deformation of the Atox1 protein.

Based on the experimental studies of the structure of a ternary cisplatin + 2Atox1 complex, the two Atox1 proteins are linked by the Pt(II) center in the asymmetric unit with two α-helical layers at the inside of the complex and two β-sheet layers at the outside [23]. It can be seen from the average structures in Figure 8 that the ligand plane of the cisplatin agent in the CisPt + 2Atox1 model almost parallels to the two α helical layers of the Atox1 dimer, and is far away from the Atox1 protein–Atox1 protein interface towards the outside of the Atox1 dimer. For the OxaliPt + 2Atox1 model, the diaminocyclohexane plane of the oxaliplatin agent is perpendicular to the two α helical layers, and is also far away from the Atox1 protein–Atox1 protein interface towards the outside of the Atox1 dimer. Such conformation with the ligand plane towards the outside of the Atox1 dimer for the two models results in the insignificant interaction between the platinum ligand and the Atox1 dimer. However, for the TransPt + 2Atox1 model, the two NH_3_ group ligands of the transplatin agent are respectively located at the outside and inside of the Atox1 dimer although the ligand plane of the transplatin agent is also perpendicular to the two α helical layers of the Atox1 dimer, which leads to the insignificant interaction between the transplatin ligand and the Atox1 dimer. These observations suggest the difference of cis and trans conformations for the cis and trans platinum agents binding to the proteins. It is evident that for the CisPt + 2Atox1 and OxaliPt + 2Atox1 models the two α helical layers of the Atox1 dimer for the cis conformations of the cisplatin and oxaliplatin agents are close to each other in the way of face-to-face with the angle of 36° between the two α1 helices. However, such layers for the trans conformation of the transplatin agent are away from each other in the way of head-to-head with the angel of 85° between the two α1 helixes, which results in the insignificant interaction between the two proteins. Moreover, to address the structural difference of cis and trans conformations for these platinum agents, the angle changes between the two proteins from the simulations for the OxaliPt + 2Atox1 and TransPt + 2Atox1 models compared with the CisPt + 2Atox1 model have been analyzed by using the DynDom program. The results suggest that the relative rotation angles of the two proteins in the OxaliPt + 2Atox1 and TransPt + 2Atox1 models are 11.6° and 70.8° (see Figure S2), respectively, compared with the CisPt + 2Atox1 model, which predicts that the trans conformation in the transplatin agent causes the larger relative rotation of the two proteins over the cis conformation for the cisplatin and oxaliplatin agents. Moreover, it can be seen from the average structures of the three models in Figure 8 that the two Atox1 proteins present an asymmetric dimeric structure in each model. For example, the obvious characteristics of the asymmetric structure are the different extent of the deviation of the α1 helix from the β2 strand. Namely, the differences of the centroid distance of five residues (Gly13-Glu17) at the α1 helix and the β2 strand between two Atox1 proteins are 0.3 Å for the CisPt + 2Atox1 model, 1.0 Å for the OxaliPt + 2Atox1 model and 1.7 Å for the TransPt + 2Atox1 model (see Figure S4a). To further investigate the structural asymmetry for these models, the interhelical angles of the same five residues at the α1 helix and the α2 helix have been quantitatively examined over the simulation times by using the program INTERHLX. The difference values of the average interhelical angles between the two proteins are 4.1°, 27.1° and 19.4° for the CisPt + 2Atox1, OxaliPt + 2Atox1 and TransPt + 2Atox1 models, respectively (see Figure S4b). Moreover, the residues Leu65 and Gly66 of the β4 strand for the CisPt + 2Atox1 model, and the residue Lys3 of β1 strand and the residue Gly13 of α1 helix for the TransPt + 2Atox1 model in the Atox1 A protein are rearranged to the loop conformations; while such conformational change is not found in the Atox1 B protein. These asymmetry structures of the two Atox1 proteins in the Atox1 dimer for the CisPt + 2Atox1 and OxaliPt + 2Atox1 models might facilitate the interactions between the two proteins, except for the TransPt + 2Atox1 model with the two proteins away from each other in the way of head-to-head. To further explain the stability of an asymmetric structure, a symmetric Atox1 dimer model has been built based on the asymmetric ternary structures of the CisPt + 2Atox1 and OxaliPt + 2Atox1 models, and is shown in Figure S5. It can be seen from analyzing the symmetric Atox1 dimer model that the original hydrogen bonds between the residue Arg21 of the α1 helix in the Atox1 A protein and the residues Gly59, Thr61 of the α2-β4 loop in the Atox1 B protein for the CisPt + 2Atox1 model, and between the residues Arg21, Gly59 of the Atox1 A protein and those of the Atox1 B protein for the OxaliPt + 2Atox1 model are destroyed; nevertheless, no new interaction was detected due to the increase of interval distance between the two proteins in the symmetric model. Moreover, based on the simulation for the new CisPt + 2Atox1 model with the platinum center binding to the two Cys12 residues of the Atox1 dimer, the similar conformational free energy of −3409 kcal/mol was obtained compared with that of −3404 kcal/mol for the original model with that binding to the two Cys15 residues. Summarily, the cis and trans conformations in the platinum agents may affect the structures of the platinum + 2Atox1 complexes. All three ternary models present the asymmetric dimeric structures for the two Atox1 proteins, which supports the experimental suggestion [23].

### 3.2. Comparison on the Structural Characteristics of the Oxaliplatin + Atox1 and Oxaliplatin + 2Atox1 Models

Based on the average structures between a platinum-bound monomeric Atox1 protein and a platinum-bound Atox1 dimer, the difference of their structural characteristics was analyzed only for the OxaliPt + Atox1 and OxaliPt + 2Atox1 models. For the OxaliPt + Atox1 model, because the ligand plane of the oxaliplatin agent is towards the α1 and α2 helices in the inside of the Atox1 protein, there exist some hydrogen bonds and hydrophobic interactions between the oxaliplatin ligand and the Atox1 protein, which contributes to stabilize the binary oxaliplatin + Atox1 complex. Nevertheless, because the ligand plane of the oxaliplatin agent for the OxaliPt + 2Atox1 model is towards the outside of the Atox1 dimer, there does not exist any significant interaction between the oxaliplatin ligand and the Atox1 dimer, whereas the considerable contributions of the interactions have been found in the Atox1 protein–Atox1 protein interface with the formations of hydrogen bonds and hydrophobic interactions. Due to the specific binding conformations of the oxaliplatin agent in the two OxaliPt + Atox1 and OxaliPt + 2Atox1 models, the binding pocket in the OxaliPt + Atox1 model caused by the oxaliplatin agent binding is larger by the size of 31.31 Å^2^/26.2 Å^3^ than that in the Atox1 A protein of the OxaliPt + 2Atox1 model. Moreover, the residues Gly13-Cys15 of the α1 helix in the protein are rearranged from the α helix structure to the loop conformations due to the oxaliplatin agent binding to the residues Cys12 and Cys15 for the OxaliPt + Atox1 model, however, such structural changes are not found for the OxaliPt + 2Atox1 model. The angles between the α1 and α2 helices, and between the β1 and β3 strands in the OxaliPt + Atox1 model are larger by the values of ~7° and ~9°, respectively, than those in the OxaliPt + 2Atox1 model. These observations indicate the oxaliplatin agent binding to the monomeric Atox1 protein induces the larger deformation of the Atox1 protein over that to the dimeric Atox1 protein. Remarkably, the oxaliplatin agent binding to either the monomeric Atox1 protein or the dimeric Atox1 protein is energetically favorable to stabilize the platinum + protein complexes, while to block its DNA binding. Moreover, it was speculated that the larger unfolding of the Atox1 protein for the oxaliplatin agent binding to Atox1 may facilitate the aggregation of the Atox1 proteins due to more unfolding residues around the binding loop region and the large binding pocket in the Atox1 protein, favoring the aggregation of another protein. It supports the previous experiment results [25].

## 4. Models and Methods

### 4.1. Initial Structures

On the basis of previous experimental studies of the Atox1 protein, the initial structure of the apo Atox1 protein (assigned as apo-Atox1 model) as the starting structure for the MD simulation was taken from the experimental structure (PDB entry 1TL5) [21] and is shown in Figure 2a. The apo Atox1 protein with the total 68 residues consists of two α helices (α1: 13–26; α2: 47–57) and four β strands (β1: 3–7, β2: 31–34, β3: 39–43, β4: 62–68). We chose the initial structures of the cisplatin, transplatin and oxaliplatin agents from the crystal structures (PDB entries 3IWL and 1PG9) [23,41] and the previous calculated results [42] (see Figure 2b). The initial structure of the transplatin agent + Atox1 protein complex (assigned as a TransPt + Atox1 model) for the MD simulation was taken from the X-ray crystal structure (PDB entry 3IWL) that presents a trans conformation of platinum center [23]. The coordinates of the platinum center in this binary model were taken from the X-ray crystal structure with the TCEP ligand and a nitrogen atom of the Cys12 residue substituted by the two NH_3_ groups. Based on the experimental suggestion on the structure of the cisplatin binding to the Atox1 protein [24,43], the initial structures of the binary CisPt + Atox1 and OxaliPt + Atox1 models used in our simulations were generated by substituting the transplatin agent of the TransPt + Atox1 model with the cisplatin and oxaliplatin agents, respectively. Similarly, for the ternary models, the initial structure of the cisplatin agent + Atox1 dimer complex (assigned as CisPt + 2Atox1 model, a dimer involves Atox1 A protein and Atox1 B protein) for the MD simulation was taken from the X-ray crystal structure of the cisplatin + 2Atox1 complex (PDB entry 3IWX) [23]. Based on the CisPt + 2Atox1 model, the initial structures of the ternary TransPt + 2Atox1 and OxaliPt + 2Atox1 models were generated by substituting the cisplatin agent with the transplatin and oxaliplatin agents, respectively. The missing residues Met1 and Glu68 in the CisPt + Atox1, TransPt + Atox1 and OxaliPt + Atox1 models, and the missing residue Met1 of an Atox1 protein in the CisPt + 2Atox1, TransPt + 2Atox1 and OxaliPt + 2Atox1 models were repaired using the loop search method in the Swiss-Pdb Viewer (http://spdbv.vital-it.ch/ [44,45]). Several different orientations of the platinum agents coordinating with the Atox1 protein for each model were chosen as the starting structures for our MD simulations, which provide a good test of whether our MD simulations are capable of driving significantly distinct starting structures to a non-distinguishable one when simulations reach equilibrium. For example, the RMSD values and the final average structures of the CisPt + Atox1 model starting from the two different initial structures predict the non-distinguishable results after the model simulations (see Figure S6a). Moreover, another construction method by docking the cisplatin agent to the Atox1 protein using the AutoDock program has also been employed to build the CisPt + Atox1 model [46]. The corresponding simulated results from the initial docked structure are similar to the original simulated one for the CisPt + Atox1 model (see Figure S6b). The 3Na^+^ and 25Cl^−^ counterions for the ternary CisPt + 2Atox1 model were added to achieve electroneutrality and to satisfy the experimental ionic strength, namely 20 mM for cisplatin agent + Atox1 dimer complex [23]. Similar counterion processes are applied to other models [47]. The systems were explicitly solvated by using the TIP3P water potential inside a rectangular box large enough to ensure the solvent shell extended to 10 Å in all directions.

### 4.2. Force Field Parameter Preparation

The atom types for the studied platinum agents, except for the platinum ions, were generated using the ANTECHAMBER module in the AMBER9 program (University of California, San Francisco, CA, USA) [48]. The electrostatic potentials of the platinum agents used for the RESP (Restrained ElectroStatic Potential) charge calculations were calculated at the B3LYP/6-31G** + LanL2DZ level [49–52] of theory using the Gaussian09 program [53]. The RESP charges of the platinum agents were derived by the RESP program based on the calculated electrostatic potentials. The force field parameters around the platinum center were referenced from the previous work because they mainly depend on the similar bond types between the platinum center and the connected atoms [54]. Other force field parameters of the platinum agents were generated from the gaff force field in the AMBER9 program.

### 4.3. Molecular Dynamics Simulation Protocols

All MD simulations were carried out using the AMBER9 package [48] and the parm99 [55,56] and gaff [57] force field parameters of AMBER. Details of the MD protocols are given in the Supplementary Information.

### 4.4. Free-Energy Analyses

The molecular mechanics Poisson-Boltzmann surface area (MM-PBSA) method [58–61] in the AMBER9 package was employed to perform the free energy analyses. The binding free energy was computed through calculating the free energy differences of ligand, receptor and their complex as follows:

(1) $$\Delta G_{\text{binding}}=G_{\text{complex}}-G_{\text{ligand}}-G_{\text{receptor}}$$

In MM-PBSA, the free energy (*G*) of each state is estimated from molecular mechanical energy *E*_MM_, solvation free energy *G*_SOLV_ and vibrational, rotational, and translational entropies *S*, respectively.

(2) $$G=E_{\text{MM}}+G_{\text{SOLV}}-TS$$

(3) $$E_{\text{MM}}=E_{\text{int}}+E_{\text{vdw}}+E_{\text{ele}}$$

(4) $$G_{\text{SOLV}}=G_{\text{pb}/\text{solv}}+G_{\text{np}/\text{solv}}$$

where *T* is the temperature; *E*_int_ is internal energy, *i.e.*, the sum of bond, angle, and dihedral energies; *E*_vdw_ is van der Waals energy; *E*_ele_ is electrostatic energy; *G*_SOLV_ is the sum of electrostatic solvation free energy, *G*_pb/solv_, and the non-polar salvation free energy, *G*_np/solv_. The entropy *S* is estimated by a normal mode analysis of the harmonic vibrational frequencies, calculated using the Nmode module in Amber9 package [62,63]. Prior to the normal mode calculations, each structure was fully minimized using a distance dependent dielectric of ɛ = 4*r* (*r* is the distance between two atoms) to mimic the solvent dielectric change from the solute to solvent until the root-mean-square of the elements of the gradient vector was less than 5 × 10^−4^ kcal·mol^−1^·Å^−1^. Then, the entropy was calculated based on standard statistical mechanics expressions [59,64]. Computational details are available in the Supplementary Information.

### 4.5. Fluctuation Analyses

The root-mean-square fluctuations (RMSF) values of residues are a measure of fluctuations and flexibility of backbone C_α_ of protein over the trajectory broken down by residues in comparison to the average structures [48,65]. RMSFi of the *C*_α_ atom of each residue was calculated as follows:

(5) $$RMSF_{i}=\sqrt{\frac{1}{T}\sum_{t=1}^{T}{\left(r_{i}(t)-\langle r_{i}\rangle \right)}^{2}}$$

where *T* is the number of snapshots considered in the time trajectory, *r_i_*(*t*), the position of the C_α_ atom of residue *i* at time *t*, and 〈 *r_i_* 〉, the time-averaged position of the C_α_ atom of residue *i*.

The dynamic feature of a protein and the extent of correlation of the motions of the different regions in a protein were assessed via the calculation of cross-correlation coefficients, *C*(*i*,*j*) given as follows:

(6) $$C(i,j)=\langle \Delta r_{i}\times \Delta r_{j}\rangle /{\left(\langle \Delta {r_{i}}^{2}\rangle \langle \Delta {r_{j}}^{2}\rangle \right)}^{1/2}$$

In the equation, Δ*r_i_* and Δ*r_j_* are the displacement vectors for atoms *i* and *j*, respectively, and the angle brackets denotes the ensemble average [48,65]. In the present study, the correlation coefficients were averaged over the regions of the protein and the resultant cross correlation coefficients are presented in the form of a two-dimensional graph. These structure analyses in the present work were calculated by using PTRAJ module in the AMBER9 program [48].

### 4.6. Calculation of Angle between Two Helices

To analyze conformational changes in the relative orientations of any two helices, the program interhlx (written by Kyoko Yap, available at (http://www.nmr.uhnres.utoronto.ca/ikura/interhlx/)) was used to calculate the angle between two helices, including the sign in a structure or a family of structures. The program calculates the sign of the angle between two helices by following this convenient role: The two helices are taken to be positioned by helix I being in front of helix II. Helix I (from N to C) is used to define first vertical vector. A second vertical vector is defined with its tail at the *C*-terminus of helix II. The angle between helices I and II is the rotation required to align the head of the second vector with the *N*-terminus of helix II. The vector is rotated in the direction that produces an angle of less than 180 degrees with the clockwise or counterclockwise rotation represented by positive or negative sign. This program can also provide other geometry-based parameters such as interhelical distances [66,67].

### 4.7. DynDom and Surface Analyses

DynDom is able to determine dynamic domains, hinge axes, and hinge-bending residues from two protein structures that have different conformations. DynDom generates short segments of the amino-acid chains of these proteins by use of a sliding window and the calculation of the rotation vector associated with the rotation of these segments between the two structures. By treating the components of these rotation vectors as coordinates in a “rotation space”, segments that rotate together will have rotation points collocated, indicating possible rigid domains within the structure. Moreover, in creating a domain decomposition, DynDom measures the ratio of interdomain displacement to intradomain displacement as defined by Hayward & Berendsen [68]. For a domain decomposition to be accepted for the hinge axis analysis, this ratio must be larger than 1.0, *i.e.*, there must be more interdomain displacement than intradomain displacement. Thus, domains can be identified from the distribution of rotation points. Domains and hinge axes were identified and characterized by using the DYNDOM program (http://www.cmp.uea.ac.uk/dyndom/).

Shape descriptors representing protein structure, such as depth, surface curvature, extreme elevation, surface area and volume, have been used extensively to identify, study and compare protein ligand interactions, protein-protein interactions and the respective binding sites. We have used CASTp server to determine the binding pocket (http://sts.bioengr.uic.edu/castp/) [69]. CASTp server uses the weighted Delaunay triangulation and the alpha complex for shape measurements. It provides identification and measurements of surface accessible pockets as well as interior inaccessible cavities, for proteins and other molecules. It measures analytically the area (in Å^2^) and volume (in Å^3^) of each pocket and cavity, both in solvent accessible surface (SA, Richards’ surface) and molecular surface (MS, Connolly’s surface) [69]. We set probe radius of 1.4 Å as default value. The detected pockets from these algorithms ranked with their volumes and areas. For ligand molecules internal protein cavities appear to be a favored binding site, and the cavity volume may play an important role in the strength of the guest molecule–host cavity interaction. In CASTp analysis, we have chosen the platinum-binding pockets for our analysis.

To analyze the influence of electrostatic properties on biochemical system, the PDB2PQR Server (http://nbcr-222.ucsd.edu/pdb2pqr_1.8/) was employed to convert the protein files in PDB format to PQR format in which the occupancy and B-factor columns have been replaced by per-atom charge and radius [70,71]. The Pymol molecular graphics software package provides support for both the evaluation of the electrostatic properties and the visualization of the resulting electrostatic potentials with PQR file.

## 5. Conclusions

Molecular dynamics simulations and free energy calculations for the constructed apo-Atox1, CisPt + Atox1, TransPt + Atox1, OxaliPt + Atox1, CisPt + 2Atox1, TransPt + 2Atox1 and OxaliPt + 2Atox1 models have been performed to address the interaction properties of the platinum agents and the Atox1 protein. All the cisplatin, transplatin and oxaliplatin agents could respectively combine with a monomeric Atox1 protein and a dimeric Atox1 protein to form a stable complex due to the covalent interaction of the platinum center of each platinum agent with the binding sites in the Atox1 protein. The calculated binding free energies for the OxaliPt + Atox1 model, except for the covalent interactions between the platinum center and the protein, suggested that the affinity of the oxaliplatin ligand with the Atox1 protein provide the extra interaction. However, such extra interaction in the CisPt + Atox1 and TransPt + Atox1 models is insignificant. The corresponding visual structure analysis proposed that the oxaliplatin agent binding to the Atox1 protein may lead to a larger unfolding and deformation of the Atox1 protein over those in the CisPt + Atox1 and TransPt + Atox1 models. The hydrogen bonds and hydrophobic interactions at the N–H/C–H groups of the oxaliplatin ligand and the residues Cys15 of the β1-α1 loop, Ala18 of the α1 helix and Thr58 of the α2-β4 loop in the Atox1 protein, and the binding pocket with the size of 82.6 Å^2^/61.5 Å^3^ around the binding sites were detected, which support the calculated affinity of the oxaliplatin agent to the Atox1 protein. For the three platinum agents binding to the Atox1 dimer, the binding free energy calculations suggest that no extra interactions come from the platinum ligand–Atox1 dimer interface except for the covalent interaction between the platinum center and the protein. However, the extra interactions between the residues Arg21, Ala18, Gly59, Thr58 of α1 helix/α2-β4 loop of the Atox1 A protein and the residues Cys15, Arg21, Gly59, Thr61, Ala18 of those of the Atox1 B protein for the CisPt + 2Atox1 and OxaliPt + Atox1 models at the Atox1 protein–Atox1 protein interface were found due to the cis conformations of the cisplatin and oxaliplatin agents. However, such extra interaction in the TransPt + Atox1 models might be insignificant due to its trans conformation. The average structure analysis speculated that the two Atox1 proteins exhibit the structural feature of the asymmetric dimers for all the three ternary models, which supports the experimental suggestion. The asymmetric structural characteristics were mainly reflected in the rearrangement of residues and the differences of the centroid distances in the two Atox1 proteins. These results might provide useful insights into understanding the interaction mechanism of platinum agents and the Atox1 protein and the experimental observations of uptake/efflux of the platinum agents in the cytoplasm.

## Supplementary Information



## Figures and Tables

**Table 1.** Molecular mechanics Poisson–Boltzmann surface area (MM-PBSA) free energy components (kcal·mol^−1^) for non-covalent interaction in the CisPt + Atox1, TransPt + Atox1 and OxaliPt + Atox1 models.

Component	CisPt + Atox1	TransPt + Atox1	OxaliPt + Atox1
Receptor	Atox1	Atox1	Atox1
Ligand	Cisplatin ligand	Transplatin ligand	Oxaliplatin ligand
Δ*E*_ele_	−46.47	11.24	−35.54
Δ*E*_vdw_	−0.64	−2.47	−9.09
Δ*E*_int_	0.00	0.00	0.00
Δ*G*_np/solv_	−8.86	−11.10	−14.93
Δ*G*_pb/solv_	45.06	−0.39	41.56
Δ*G*_np_	−9.50	−13.57	−24.02
Δ*G*_pb_	−1.41	10.85	6.02
Δ*H*_binding_	−10.91	−2.73	−18.00
*T*Δ*S*	−16.19	−15.14	−13.87
Δ*G*_binding_	5.28	12.41	−4.13

Δ*G*_pb_ = Δ*E*_ele_ + Δ*G*_pb/solv_; Δ*G*_np_ = Δ*E*_vdw_ + Δ*G*_np/solv_; Δ*G*_binding_ = Δ*G*_np_ + Δ*G*_pb_ + Δ*E*_int_ − Δ*TS* = ΔH_binding_ – Δ*TS*.

**Table 2.** The occupancies (%) of the hydrogen bonds and hydrophobic interactions between the oxaliplatin ligand and the Atox1 protein for the OxaliPt + Atox1 model.

Hydrogen bond	OxaliPt + Atox1
(Cys15)CB H–N1	99.0
(Cys15)CA H–N2	77.6
N1 H–CA(Cys15)	30.6

**Hydrophobic contact**	

(Val8)CG1 C4(oxaliplatin)	65.4
(Val8)CG1 C5(oxaliplatin)	88.7
(Val8)CG1 C3(oxaliplatin)	47.2
(Asp9)CB C5(oxaliplatin)	90.3
(Cys15)CA/CB C1(oxaliplatin)	197.2
(Ala18)CB C2(oxaliplatin)	76.3
(Thr58)CB C4(oxaliplatin)	50.9
(Thr58)CB/CG2 C3(oxaliplatin)	126.5
