# Supplementary material for: Interaction of Classical Platinum Agents with the Monomeric and Dimeric Atox1 Proteins: A Molecular Dynamics Simulation Study
Source: Int J Mol Sci. 2013 Dec 20;15(1):75–99. doi: 10.3390/ijms15010075 (PMC3907799; doi:10.3390/ijms15010075)
Supplement: Supplementary file 1 [file ijms-15-00075-s001.pdf]

Supplementary Information

1. Molecular Dynamics Simulation Protocols

All MD simulations were carried out using the AMBER9 package [48] with a classical AMBER parm99 [55,56] together with the gaff [72] force field parameters. The protocol for all MD simulations is described herein as follows: (1) the systems were energetically minimized to remove unfavorable contacts. Four cycles of minimizations were performed with 5000 steps of each minimization and harmonic restraints on the platinum agents and the Atox1 protein from 100, 75, 50 to 25 kcal·mol^{-1}·Å^{-2}, which means that the restraints were relaxed stepwisely by 25 kcal·mol^{-1}·Å^{-2} per cycle. The fifth cycle consists of 10,000 steps of unrestrained minimization before heating process. The cutoff distance used for the non-bonded interactions was 10 Å. The SHAKE algorithm [73] was used to restrain the bonds containing hydrogen atoms; (2) Each energy-minimized structure was heated over 120 ps from 0 to 300 K (with a temperature coupling of 0.2 ps), while the positions of the platinum agents and the Atox1 protein were restrained with a small value of 25 kcal·mol^{-1}·Å^{-2}. The constant volume was maintained during the processes; (3) The unrestrained equilibration of 200 ps with constant pressure and temperature conditions was carried out for each model. The temperature and pressure were allowed to fluctuate around 300 K and 1 bar, respectively, with the corresponding coupling of 0.2 ps. For each simulation, an integration step of 2 fs was used; (4) Finally, production runs of 50 ns were carried out by following the same protocol. A time point after thermal equilibration of 200 ps in each simulation was selected as a starting point for data collection. During the production runs, 15,000–25,000 structures for a simulation were saved for post-processing by uniformly sampling the trajectory.

2. MM-PBSA Calculation for Free Energy

Energetic post-process was performed for each MM-PBSA calculation by using the MM-PBSA module of AMBER9 program through molecular mechanics and a continuum solvent model [48]. In MM-PBSA calculation, $G_{\mathrm{np/solv}}$ is non-polar solvation free energy, which was calculated by using a solvent accessible surface area (SASA) as follows:

$$G_{\mathrm{np/solv}} = rSASA + b \quad \text{(S1)}$$

The *SASA* is the solvent-accessible surface area, and is estimated using Sanner's algorithm implemented in the Molsurf program in AMBER9 [74] with a probe radius of 1.4 Å. The surface tension proportionality constant (r) and the free energy of non-polar solvation for a point solute (b) are set to 0.04356 kcal mol^{-1} Å^{-2} and −1.008 kcal mol^{-1}, respectively.

For each model, the last 20 ns trajectory of the production dynamics stage was used for binding free energy calculations of MM-PBSA, namely, the 5000 snapshots of each model at a 4-ps interval for computation of enthalpy and 20 snapshots at 1000-ps intervals for computation of entropy. Because each platinum ion of the three agents forms two same covalent bonds with two same residues of the protein, such equivalent covalent interactions were ignored by stripping the platinum ion from each trajectory in the binding free energy calculations.

Figure S1. Conformational stability free energies (kcal·mol^{-1}) and integrated distributions for (**a**) the CisPt + Atox1 model (**green**); (**b**) the TransPt + Atox1 model (**magenta**); and (**c**) the OxaliPt + Atox1 model (**cyan**).

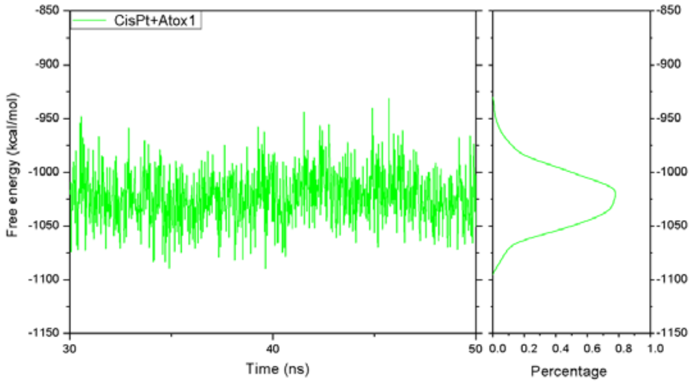

(**a**)

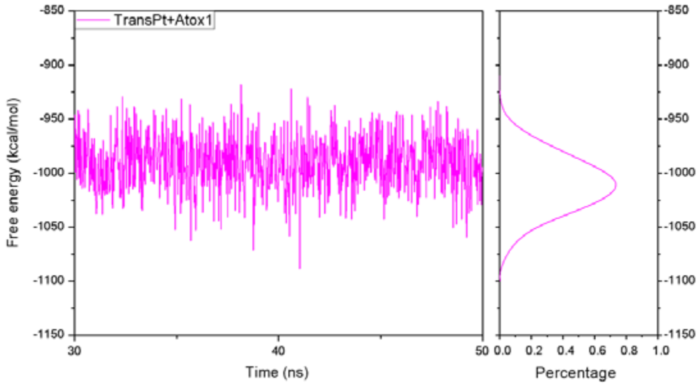

(**b**)

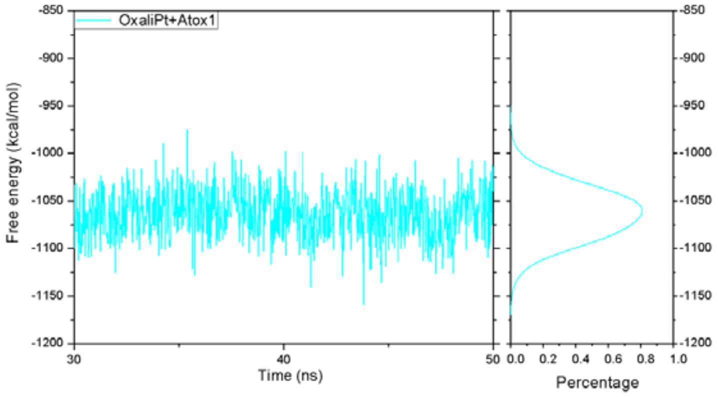

(**c**)

Figure S2. The relative rotation angle of the two dimeric Atox1 proteins between the CisPt + 2Atox1 (**light blue**) and TransPt + 2Atox1 (**cyan**) models.

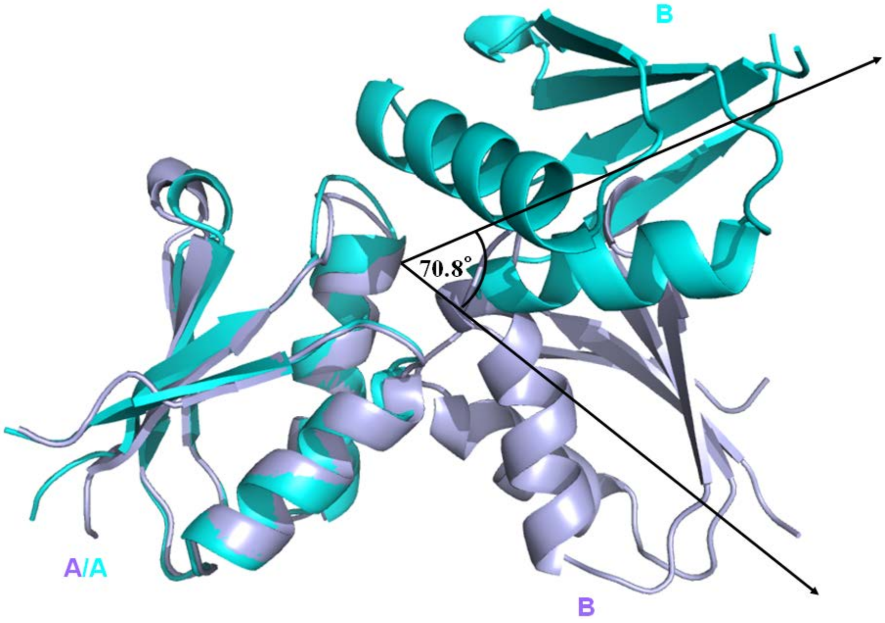

Figure S3. The scheme of the hydrogen bond and hydrophobic interactions between the Atox1 A and Atox1 B proteins for the CisPt + 2Atox1 and OxaliPt + 2Atox1 models (N: deep blue; C: cyan; S: yellow; O: red; H: white).

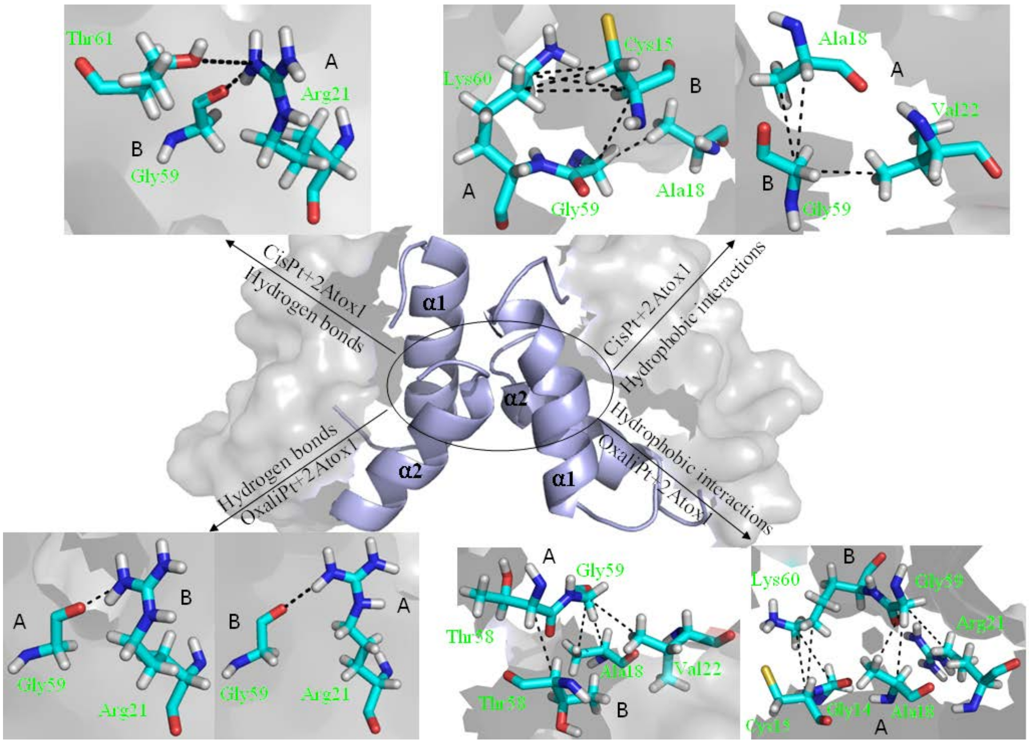

Figure S4. (**a**) The time-dependences of the centroid distances between the residues Gly13-Glu17 of the α1 helix and the β2 strand for the Atox1 A protein (**light blue**) and the Atox1 B protein (**cyan**) in the TransPt + 2Atox1 model. The inset is the visual superposition for the average structures of the α1 helix and the β2 strand for the two proteins; and (**b**) The time-dependence of the angles between the residues Gly13-Glu17 of the α1 helix and the α2 helix for the Atox1 A protein (**light blue**) and the Atox1 B protein (**cyan**) in the TransPt + 2Atox1 model. The inset is the visual superposition for the average structures of the α1 helix and the α2 helix for the two proteins.

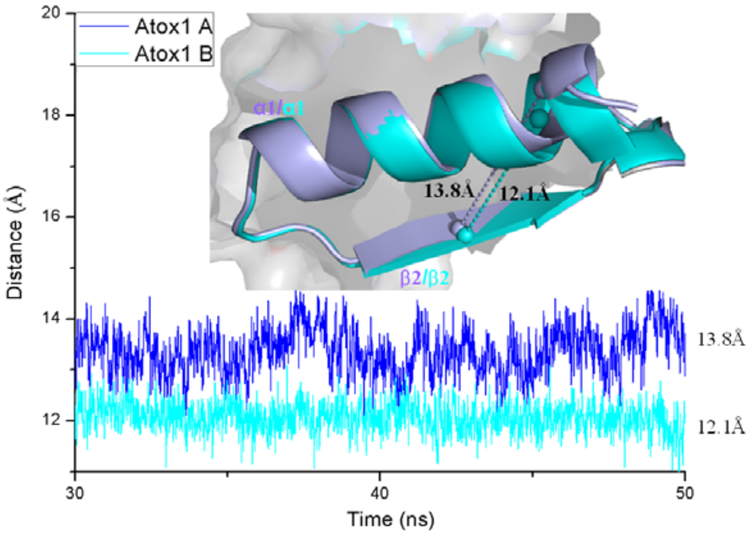

(**a**)

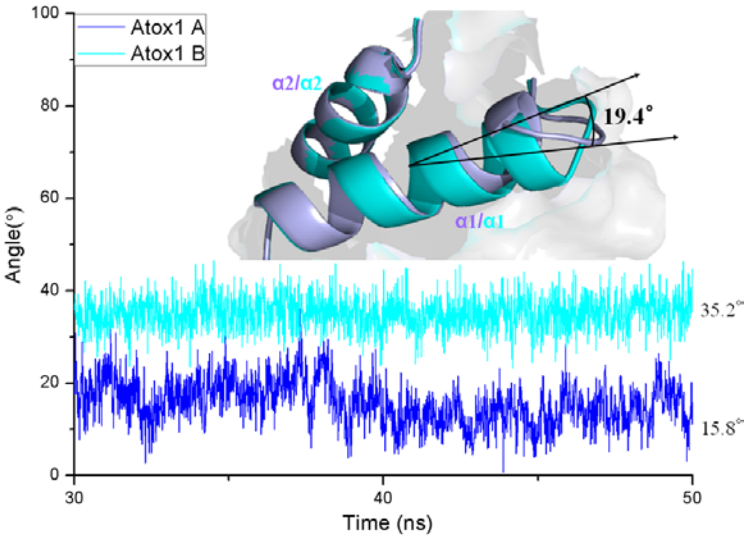

(**b**)

Figure S5. The visual superposition for the asymmetric structure (**white**) based on the average structures of the CisPt + 2Atox1 and OxaliPt + 2Atox1 models and the symmetric structure (**light blue**).

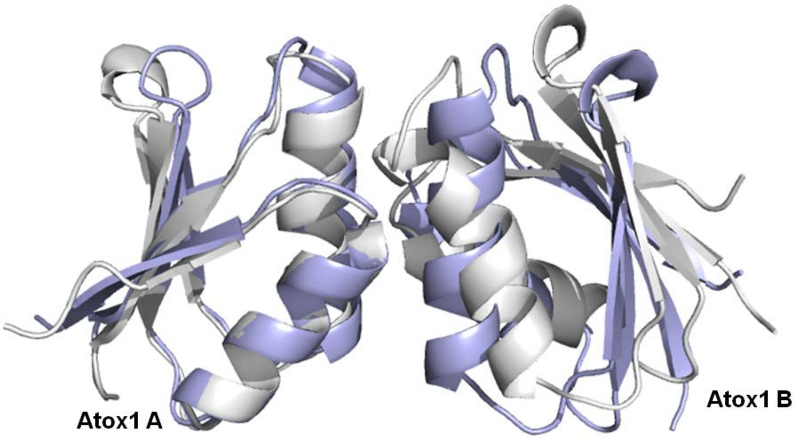

Figure S6. RMSD values of all backbone atoms and the final average structures with respect to the different starting structures for the CisPt + Atox1 model built by (**a**) the original substituting method (**light blue** and **cyan**); and (**b**) the docking method and the original one (**green** and **light blue**).

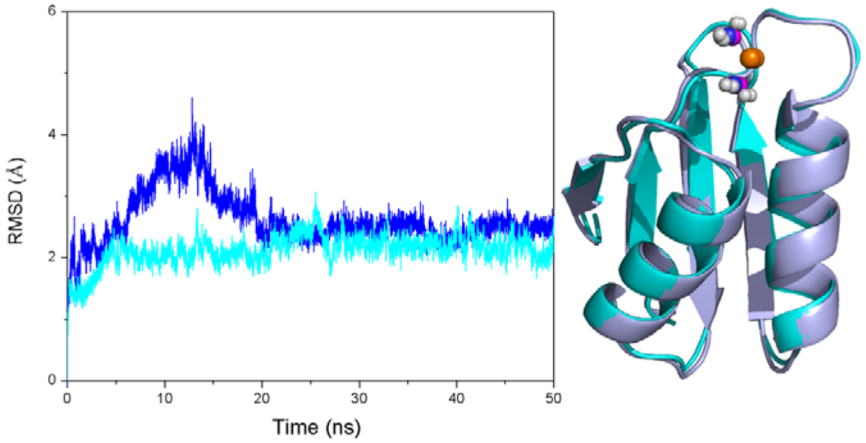

(**a**)

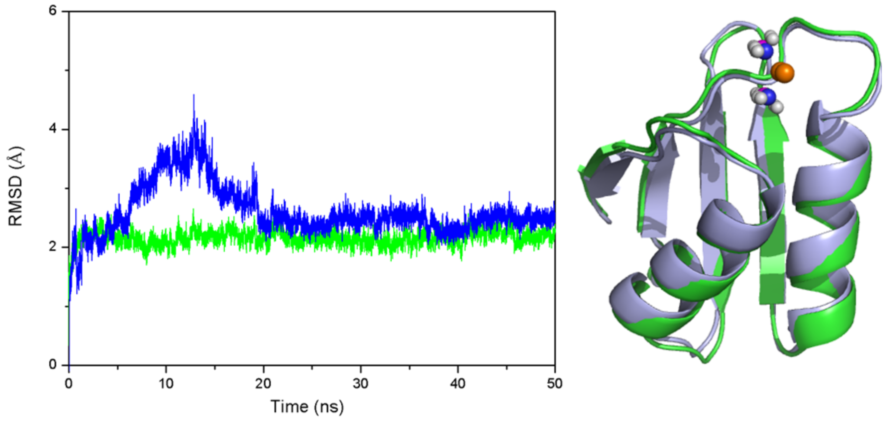

(**b**)

Table S1. MM-PBSA free energy components (kcal·mol^{-1}) for non-covalent interaction in the CisPt + 2Atox1, TransPt + 2Atox1, OxaliPt + 2Atox1 models.

**Component**	**CisPt + 2Atox1**	**TransPt + 2Atox1**	**OxaliPt + 2Atox1**	**CisPt + 2Atox1**	**TransPt + 2Atox1**	**OxaliPt + 2Atox1**
Receptor	2Atox1	2Atox1	2Atox1	Atox1	Atox1	Atox1
Ligand	Cisplatin ligand	Transplatin ligand	Oxaliplatin ligand	Atox1	Atox1	Atox1
ΔE_{ele}	35.5	35.83	17.66	58.70	26.51	66.94
ΔE_{vdw}	−2.28	−1.47	−11.07	−36.73	−16.63	−39.19
ΔE_{int}	0.00	0.00	0.00	0.00	0.00	0.00
$\Delta G_{np/solv}$	−10.08	−12.13	−15.60	−33.59	−21.39	−37.13
$\Delta G_{pb/solv}$	−17.97	−10.28	7.73	−19.43	−4.26	−23.39
ΔG_{np}	−12.38	−13.60	−26.67	−70.32	−38.02	−62.58
ΔG_{pb}	17.58	25.55	25.38	39.26	22.25	43.55
$\Delta H_{binding}$	5.22	11.95	−1.29	−31.06	−15.78	−32.77
$T\Delta S$	−14.58	−14.92	−13.26	−27.49	−23.12	−27.71
$\Delta G_{binding}$	19.8	26.87	14.55	−3.57	8.65	−5.06

$\Delta G_{pb} = \Delta E_{ele} + \Delta G_{pb/solv}$ $\Delta G_{np} = \Delta E_{vdw} + \Delta G_{np/solv}$ $\Delta G_{binding} = \Delta G_{np} + \Delta G_{pb} + \Delta E_{int} - \Delta TS = \Delta H_{binding} - \Delta TS$.

Table S2. The occupancies (%) of hydrogen bond and hydrophobic interactions between the Atox1 A protein and Atox1 B protein for the CisPt + 2Atox1 and OxaliPt + 2Atox1 models.

	**Hydrogen bond**		**CisPt + 2Atox1**	**OxaliPt + 2Atox1**
Atox1 A	(Arg21)NH–H O(Gly59)	Atox1 B	75.6	62.2
	(Arg21)NH–H OG1(Thr61)		13.6	
	(Gly59)O H–NH1(Arg21)			85.7
	**Hydrophobic contact**			
Atox1 A	(Gly59)CA CA(Cys15)	Atox1 B	72.6	
	(Lys60)CE CA/CB(Cys15)		138.1	
	(Lys60)CD CA/CB(Cys15)		135.3	
	(Gly59)CA (Ala18)CA/CB		96.9	166.2
	(Gly59)CA CG1(Val22)			80.7
	(Thr58)CA CA(Thr58)			97.6
	(Ala18)CA/CB CA(Gly59)		172.7	175.7
	(Arg21)CD CA(Gly59)			43.1
	(Val22)CC1 CA(Gly59)		88.3	
	(Cys15)CA CD/CE(Lys60)			142.8
	(Gly14)CA·CE(Lys60)			61.1

© 2013 by the authors; licensee MDPI, Basel, Switzerland. This article is an open access article distributed under the terms and conditions of the Creative Commons Attribution license (http://creativecommons.org/licenses/by/3.0/).
